# Metabolic Effects of the Intracellular Regulation of Thyroid Hormone: Old Players, New Concepts

**DOI:** 10.3389/fendo.2018.00474

**Published:** 2018-09-11

**Authors:** Annunziata G. Cicatiello, Daniela Di Girolamo, Monica Dentice

**Affiliations:** Department of Clinical Medicine and Surgery, University of Naples Federico II, Naples, Italy

**Keywords:** Thyroid hormone, Deiodinases, energy metabolism, Thyroid hormone receptors, local regulation of thyroid function

## Abstract

Thyroid hormones (THs) are key determinants of cellular metabolism and regulate a variety of pathways that are involved in the metabolism of carbohydrates, lipids and proteins in several target tissues. Notably, hyperthyroidism induces a hyper-metabolic state characterized by increased resting energy expenditure, reduced cholesterol levels, increased lipolysis and gluconeogenesis followed by weight loss, whereas hypothyroidism induces a hypo-metabolic state characterized by reduced energy expenditure, increased cholesterol levels, reduced lipolysis and gluconeogenesis followed by weight gain. Thyroid hormone is also a key regulator of mitochondria respiration and biogenesis. Besides mirroring systemic TH concentrations, the intracellular availability of TH is potently regulated in target cells by a mechanism of activation/inactivation catalyzed by three seleno-proteins: type 1 and type 2 iodothyronine deiodinase (D1 and D2) that convert the biologically inactive precursor thyroxine T4 into T3, and type 3 iodothyronine deiodinase (D3) that inactivates TH action. Thus, the pleiotropic effects of TH can fluctuate among tissues and strictly depend on the cell-autonomous action of the deiodinases. Here we review the mechanisms of TH action that mediate metabolic regulation. This review traces the critical impact of peripheral regulation of TH by the deiodinases on the pathways that regulate energy metabolism and the balance among energy intake, expenditure and storage in specific target tissues.

## Introduction

Thyroid hormones (TH) have long been known to regulate energy metabolism (1). Patients with TH dysfunction often have symptoms of metabolic dysregulation, including fatigue and weight changes (2). Indeed, pathological excess of THs in humans raises the basal metabolic rate (BMR) while TH deficiency is accompanied by a decreased BMR (2). TSH and TRH levels are also critical determinants of whole body energy metabolism. In fact, they exert thyroidal and non-thyroidal effects and thus integrate signals from nutritional status and the adrenergic nervous system with a fine regulation of THs production (3). The wide spectrum of THs effects on body metabolism is exerted mainly by stimulating catabolic and anabolic reactions and by regulating turnover of fats, carbohydrates and proteins (1). A peculiar feature of TH-dependent metabolic regulation is the acceleration of the rates of anabolic and catabolic reactions (4). For instance, TH increases fat mobilization thereby leading to increased concentrations of fatty acids in plasma as well as to enhanced oxidation of fatty acids. THs stimulate insulin-dependent glucose uptake, and both gluconeogenesis and glycogenolysis. Therefore, the action of THs culminates in promoting futile cycles that contribute significantly to the increase oxygen consumption seen in thyrotoxicosis (“hyperthyroidism”). Thyroid hormones also stimulate ion cycling by altering membrane permeability, the expression of ion pumps and the characteristics of these pumps (5–8).

The classic endocrine view of TH biology is that THs are produced and secreted by the thyroid gland for transport to target tissues. Accordingly, TH concentrations determine the extent of hormonal regulation and generate downstream effects in peripheral cells. Classical regulation of the thyroid gland involves the hypothalamic–pituitary–thyroid axis, whereas low TH concentrations trigger a negative feedback that results in the release of both thyroid releasing hormone (TRH) from the hypothalamus and thyroid stimulating hormone (TSH) from the pituitary gland (9, 10). However, besides the capacity of the thyroid gland to produce the correct amount of THs, the periphery can modify the TH signal in time and space. Indeed, while plasma concentrations of TH are relatively stable, tissues can coordinate TH levels through the cell-autonomous regulation of TH transporters, deiodinases and TH receptors (11). The iodothyronine deiodinase family of selenoproteins is constituted by three enzymes, D1, D2, and D3. These enzymes are present in specific tissues, and regulate TH activation and inactivation (12). The differential expression of deiodinases enables close control of T3 and its prohormone, T4, by removing iodine moieties (“deiodination”) at different sites of the phenolic or tyrosylic ring of the TH hormones (13). T4 has a long half-life and is converted to the active form, T3, within cells by the activating deiodinases (D1 and D2) that catalyze outer ring deiodination. The third deiodinase, D3, terminates TH action by inactivating T3 and T4 by removing the iodine at the inner ring (13). The local regulation of TH at intracellular level enables wide fluctuations of TH in local tissues and is a powerful tool with which to modulate TH action without perturbing systemic TH levels.

The correlation of a Thr92Ala polymorphism in the DIO2 gene, encoding protein D2, with altered glycemic control, obesity and type 2 diabetes mellitus (T2DM) (14–16), as well as the association of genetic variants of the DIO1 gene, encoding protein D1, with insulin resistance (17), reinforces the clinical relevance of the peripheral T4-to-T3 conversion in metabolic control.

In this review we summarize the role of the local control of TH by the deiodinases in the metabolic program of cells in the context of the tissue-specific impact of deiodination on energy metabolism, and discuss the effect of local alteration of TH on the metabolic functions of the body.

## Metabolic role of TH and the deiodinases

**Figure 1:**
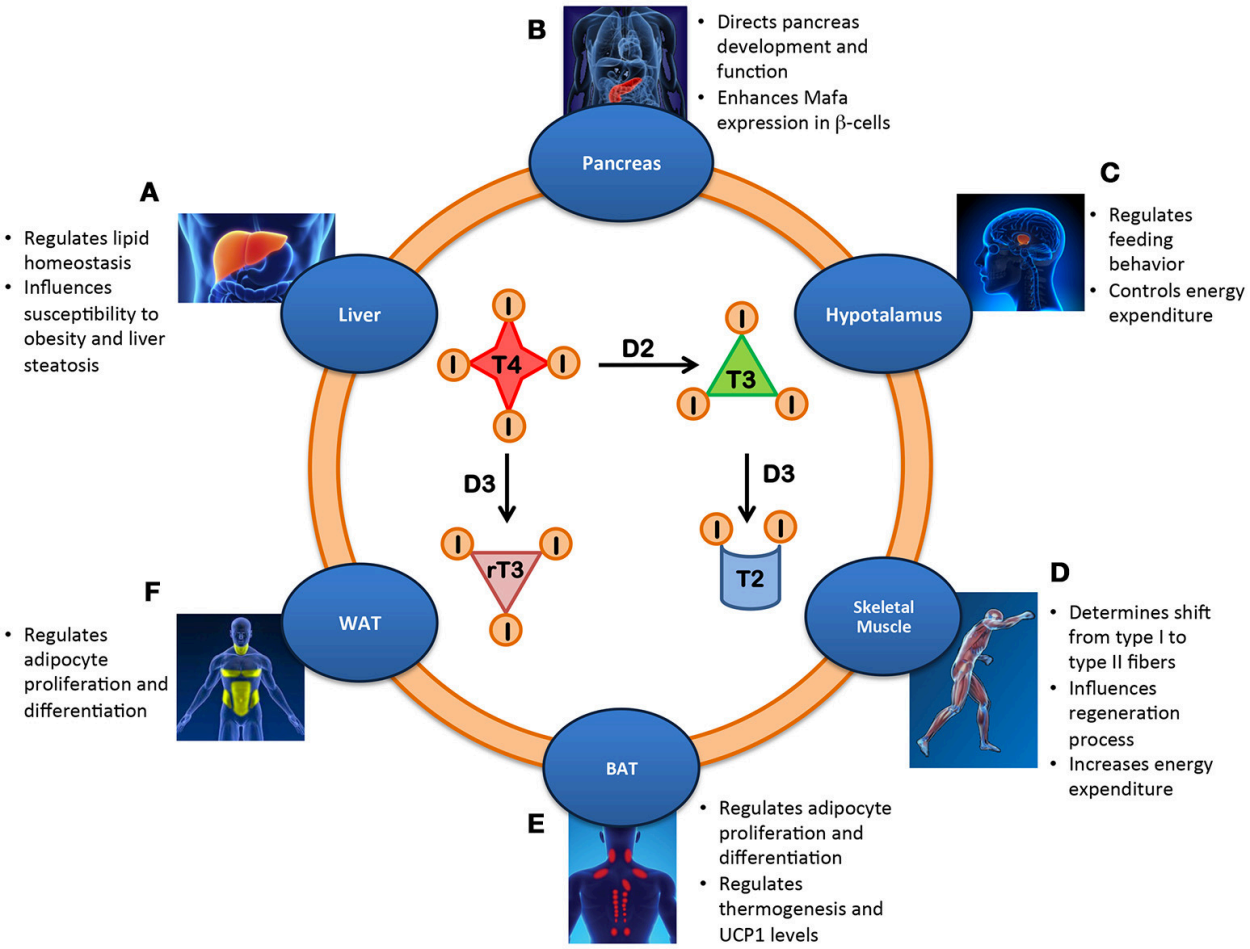
Metabolic effects of the intracellular regulation of thyroid hormone in different tissues. **(A)** Liver: Lipid homeostasis is regulated by local T3 level thereby influencing susceptibility to obesity and liver steatosis. **(B)** Pancreas: The balance between the deiodinases controls the development and function of β-cells by enhancing Mafa transcription factor and inducing insulin secretion. **(C)** Hypothalamus: Local availability of TH regulates feeding behavior and controls energy expenditure. **(D)** Skeletal Muscle: Increased T3 levels in skeletal muscle promote the shift from type I to type II fibers, influence regeneration and increase energy expenditure. **(E)** BAT: D2-mediated TH activation regulates UCP1 expression and thermogenesis, adipocyte proliferation and differentiation and body weight. **(F)** WAT: Local T3 metabolism regulates adipocyte proliferation/differentiation.

Although each cell of the body is virtually a TH target, the TH signal is differentially integrated in each tissue depending on the cell-autonomous machinery. Therefore, the action of TH on whole body metabolism is best evaluated by examining the specific contribution of TH and its modulating enzymes to energy metabolism in the context of each target tissue. The relative roles of most components of the TH signaling pathways have been assessed in mouse models of inducible, tissue-specific activation or inactivation of deiodinases, receptors and transporters (1). These studies revealed how different TH-induced processes contribute to regulating metabolic homeostasis in humans (Figure 1).

## Liver

There is an intricate relationship between TH metabolism and liver (1, 18, 19). Thyroid hormones regulate hepatic function by modulating the basal metabolic rate of hepatocytes; the liver in turn metabolizes the THs and regulates their systemic endocrine effects (20). In the liver, TH regulates lipid metabolism mainly through the T3-TRβ (TH receptor beta) (1), and the downstream regulation of cholesterol homeostasis (synthesis and efflux), bile acid synthesis and fatty acid metabolism (1). The local control of TH metabolism in liver is mediated by the expression of all three deiodinases. D1 is highly expressed in liver, where it contributes to plasmatic T3 homeostasis and mediates the clearance of rT3 from the circulation (21). D1 expression is highly sensitive to T3 levels to such an extent that it is an indicator of the thyroid state of the liver (22). Despite the high D1 levels in the liver, the intracellular T3 level in hepatocytes is not mediated by D1, but by the other TH-activating deiodinase, D2 (23). The liver is the paradigm of spatio-temporal regulated expression of D2 that is transiently turned on in the neonatal mouse liver between the first and the fifth post-natal day. During this short time, a peak of hepatic D2 expression occurs and rapidly declines to background levels (24). This brief peak of D2 produces an excess of T3 that modifies the methylation and the expression pattern of thousands of hepatic genes thereby increasing future susceptibility to diet-induced obesity and liver steatosis (24). Mouse models of hepatocyte-specific D2 inactivation (Alb-D2KO) do not undergo this physiological increase of liver T3 at birth, with a consequent delay in neonatal expression of lipid-related genes and a phenotype of resistance to obesity and liver steatosis (24). These fundamental changes during perinatal life indicate that the thyroid state of specific tissue impacts on whole body metabolism thereby affecting the phenotype in adult life (25). Finally, D3 is almost undetectable in the liver of healthy individuals, but robust re-activation of D3 has been found in regenerating liver tissue, in certain hepatic tumors and in sera and liver samples from critically ill humans, thus influencing the systemic thyroid status (26). These results suggest that D3 plays a role in the tissue response to injury and in the imbalance of TH homeostasis commonly observed during critical illness.

## Pancreas

Thyroid hormone plays a critical role in the development, maturation, and function of pancreatic cells, where T3 is required for the physiological maturation of pancreatic β-cells to glucose-stimulated insulin-secreting cells (27). Pancreatic cells express both TRα and TRβ isoforms and the activated T3-TR complex directly bind to the promoter of islet transcription factor Mafa thereby resulting in its activation (27, 28). However, the exact physiological role of TH in glucose homeostasis remains controversial (29, 30). Although numerous *in vitro* and *ex vivo* studies have demonstrated that T3 mediates positive effects on β-cell function, exposure to high doses of TH results in a phenotype of glucose intolerance. Indeed, hyperthyroidism is associated with glucose intolerance consequent to decreased insulin secretion (31, 32) and to stimulation of hepatic gluconeogenesis (33). Probably, in hyperthyroid conditions, impaired insulin secretion is not sufficient to suppress high hepatic glucose production. Accordingly, the prevalence of diabetes mellitus in hyperthyroid patients is approximately double that of non-affected subjects (34). In contrast, systemic hypothyroidism is associated with reduced hepatic gluconeogenesis and enhanced insulin sensitivity, as demonstrated by the onset of a hypoglycemic state after an insulin injection (35). While during vertebrate development, reduced TH levels are important for normal function and for glucose homeostasis of pancreatic β-cells, exposure to high TH doses induces apoptosis of pancreatic β-cells (36). In this context, the TH hormone-inactivating deiodinase D3 plays a fundamental role in lineage fate decisions and endocrine cell specification (34). Indeed, studies in D3KO mice demonstrated that the reduction D3-mediated of TH action is critical for normal maturation and function of pancreatic β-cells (34). D3KO mice exhibited a glucose intolerant phenotype due to impaired glucose-stimulated insulin secretion, reduced size, and absolute mass of pancreatic islet and β-cells, decreased insulin content, and reduced expression of key genes involved in glucose sensing, insulin synthesis, and exocytosis (34). The pancreatic phenotype of the D3KO mice is proof that attenuation of TH-signaling via D3 activation is essential for normal development.

## Hypothalamus

Peripheral TH signals are integrated within the hypothalamus and processed into coordinated responses to regulate energy balance. The center for regulation of food intake and of body weight is the melanocortin system, constituted by three neuronal populations: the pro-opiomelanocortin (POMC)-expressing neurons, the neuropeptide Y (NPY) and agouti-related peptide (AgRP)-co-expressing neurons and the melanocortin 4 receptor (MC4R)-expressing neurons (37, 38). The POMC neurons exert an anorexigenic function by activating MC4R neurons, which induce a reduction of food intake and increased energy expenditure. On contrary, NPY/AgRP neurons are the orexigenic neurons: by antagonizing the action exerted by POMC on MC4R, they increase food intake and decrease energy expenditure. All these neurons are sensitive to the TH signal that can either activate or inhibit melanocortin neurons, and thus, it is not surprising that local TH metabolism plays a critical role in appetite and feeding regulation. Changes in central T3 levels occur in various metabolic conditions (39), for example elevated T3 levels have been found in the hypothalamus during fasting (40). Fasting induces alterations in the thyroid state, namely, a reduction in pituitary D2 levels and liver D1 levels correlated with low peripheral T3 levels in the presence of increased hypothalamic D2 activity. The high D2 activity in the hypothalamus causes an increase of local T3 concentrations, which in turn activate orexigenic NPY/AgRP neurons and inhibit anorexigenic POMC neurons, thereby inducing hyperfagia (1). The molecular mechanism underlying TH-mediated NPY/AgRP activation resembles that in brown adipose tissue (BAT) in which T3 increases uncoupling protein 1 (UCP1) activity. In fact, high T3 levels in the hypothalamus during fasting, consequent to D2 activation, promote UCP2 expression and stimulate mitochondrial proliferation in orexigenic NPY/AgRP neurons, so promoting their activity and stimulating rebound feeding upon food deprivation. The increase of T3 in the hypothalamus also causes TRH mRNA suppression (40, 41). Therefore, under food deprivation, despite a reduction in peripheral TH levels, there is a localized increase in T3 within the hypothalamus, which in turn increases orexigenic signals and decreases TRH production. The hypothalamus probably maintains low TH levels to preserve energy stores, which would be dissipated in hyperthyroid condition.

The fundamental role of deiodinases in the regulation of energy balance in brain has been demonstrated in mouse models of deiodinases depletion (42). Despite the low TH circulating levels in adult *Dio3*^−/−^ mice, their central nervous system is in a hyperthyroid state (42). The enhanced TH levels alter the functioning of the hypothalamic circuitries, including the leptin-melanocortin system, thereby regulating energy balance and adiposity. In detail, *Dio3*^−/−^ mice have decreased adiposity, but an abnormally functioning leptin-melanocortin system associated with leptin resistance (43). The hypothalamic D2-mediated T4 to T3 conversion is important for the photoperiodic response of the gonads (44) in which fine-tuned D2 and D3 expression tightly regulates LH stimulation (45).

## Skeletal muscle

Skeletal muscle represents 40–50% of the total body mass in humans and is crucial for metabolism, heat generation and maintenance of posture. TH influences skeletal muscle contraction, regeneration and metabolism (46). All components of the TH signaling process, from TR to TH transporters (MCT8 and MCT10), and D2 and D3, are expressed in the skeletal muscle of rodents and humans (47). During skeletal muscle development, D2 is up-regulated, particularly during the first postnatal days, and decreases at day 30, although its activity returns to high levels during differentiation of muscle stem cells (12, 48, 49). In particular, during post-injury regeneration processes, D2 mRNA is up-regulated to enable correct myoblast differentiation (50). D2 is a target of FOXO3, which is a protein involved in myocyte fusion and metabolism as well as in atrophy and autophagy (12). Loss of D2 impairs stem cell differentiation and prevents up-regulation of myogenic transcription factor MyoD thereby increasing the proliferative potential of muscle stem cells. D2-mediated TH in skeletal muscle influences also muscle fibers. High TH levels induce a shift from type I fibers (slow) to type II fibers (fast), which results in up-regulation of sarcoendoplasmic reticulum Ca2^+^ATPase, of glucose transporter 4 (GLUT4) and of uncoupling protein 3 (UCP3) thereby producing heat and increasing energy expenditure (51). D2-dependent T3 activation influences insulin response in skeletal muscle (52). Indeed, D2KO mice are insulin-resistant, which demonstrates the relevance of D2 in glucose homeostasis. In humans, a common polymorphism of the *Dio2* gene, the Thr92Ala substitution in protein D2, which partially impairs enzymatic activity, has been correlated with insulin resistance and diabetes (53, 54). Furthermore, muscle fibers respond to cold through TH-related mechanisms, namely increased glucose uptake, activation of oxidative pathways and increased mitochondria biogenesis (55, 56). Interestingly, D2 is up-regulated in muscle after 4 h of cold exposure (57). Moreover, D2 is up-regulated in response to such metabolic signals as bile acids and insulin (1, 58) and during exercise under β-adrenergic stimulus in order to amplify TH signaling and regulate PGC-1α expression (59, 60). Coordinated D2-D3 expression is required to fine-tune intracellular TH availability during muscle stem cell differentiation, and *in vivo*, during muscle regeneration (47). While D2 is essential for a correct T3 surge and the subsequent differentiation of muscle stem cells, D3 fosters muscle stem cell proliferation by lowering TH availability during the early phases of the myogenic program (47). This dual regulation is so critical that D3-depletion *in vivo* causes massive apoptosis of proliferating satellite cells and drastically impairs a full regeneration process. These studies highlight the pivotal role of the intracellular TH coordination by the deiodinases in muscle physiology.

## Brown adipose tissue

Brown adipose tissue is characterized by multilocular lipid droplets and numerous mitochondria, and governs heat production (61). In fact, BAT is activated in response to a high fat diet or cold exposure in order to protect the organism from weight gain and hypothermia. Thyroid hormone critically influences BAT activity (62). The most obvious metabolic role of D2 is the regulation of energy expenditure in the BAT of small mammals, including human newborns. During cold exposure, the sympathetic nervous system induces D2 expression in brown adipocytes, thereby promoting local T4-to-T3 conversion, and activation of the transcription of target genes involved in the thermogenic program (63). Loss of function of D2 reduces the level of UCP-1, which is normally up-regulated at RNA level by TH. D2 is thus considered a marker of BAT activity (1, 57). Interestingly, global D2KO mice are resistant to diet-induced obesity, highly tolerant to glucose, and have a deficit in respiratory quotient at 22°C, while at 30°C they become more susceptible to obesity and develop intolerance to glucose (64, 65). T3 regulates the expression of several genes during adipogenic differentiation, among which GPD, ME, PEPCK, S14, FAS, and GLUT4 (66, 67). While D2 activity is important during differentiation, D3 is considered a mitogenic marker in brown pre-adipocytes. In fact, D3 mRNA and activity are induced by bFGF and aFGF in proliferating brown pre-adipocytes (68). In BAT, T3 also accelerates fatty acid oxidation and lipogenesis through the action of the ACC and ME lipogenic enzymes. Consequently, D2KO mice have reduced fatty acid oxidation and lipogenesis (4).

## White adipose tissue

The primary function of white adipose tissue (WAT) is to store energy in the form of single large lipid droplets, although it also secretes the leptin and adiponectin adipokines. White adipocytes differ anatomically and physiologically from brown adipocytes. However, the latter may appear at sites corresponding to WAT, in a so-called process of WAT “browning” caused by a thermogenic stimulus, such as prolonged cold exposure (69), or treatment with β_3_-adrenergic receptor activators (70). The brown adipocytes in WAT are often called “inducible,” “beige,” or “brite.” D1 and D2 are barely expressed in epidermal WAT, the adipose tissue that, contrary to inguinal WAT, is never converted in BAT. All TR isoforms and the TH transporter MCT8 are expressed in human subcutaneous adipose tissue (71). D1 expression in epidermal WAT is only 1% of the D1 found in the liver. Similarly, the D2 mRNA level is 7% of the D2 in BAT (72). Interestingly, D1 expression and activity are increased in the subcutaneous and visceral WAT of obese subjects (71). On the other hand, D2 is up-regulated in beige/brite adipocytes and its expression is correlated to increased energy expenditure (73). A high-fat diet stimulates D1 and leptin expression, while caloric restriction decreases D1 activity as well as leptin levels, and increases levels of the leptin mediator SCD-1. Leptin overexpression increases D1 activity and down-regulates SCD-1 expression (74). Similar to brown adipocytes, in white adipocytes, D2 plays an important role in lipogenesis and in the regulation of the expression of genes related to adipocyte differentiation, while D3 sustains the proliferation of white adipocytes (61). Interestingly, thyroidectomized mice have an increased level of both D1 and D2 (72). Moreover, D2 is expressed also in human pre-adipocytes although its role is unclear (75).

## Future directions and conclusions

Monodeiodination is quantitatively the most important pathway of TH activation. Within peripheral tissue, multiple pathways modulate TH availability. These pathways govern the action and regulation of deiodinase expression, the action of TH transporters, and the expression and crosstalk of TH receptors with multiple partners. This intricate network of TH modifiers increases the sensitivity and the speed of responses to changes induced in the internal and external environment by the thyroid signal. The price to be paid for this is an intricate regulation of each component in time and space. Given the vast spectrum of metabolic body functions regulated by the TH signal, the deiodinases represent a powerful tool with which to modulate cellular metabolism in specific tissues without perturbing systemic levels of THs. Consequently, the development of drugs that target deiodinase action is the next challenge in this field. Extensive work is still required to delineate the kinetics and regulation of the deiodinase enzymes in specific tissues to understand the full spectrum of their biological roles. Thus, pharmacological research is poised to develop deiodinase modulators aimed at driving specific metabolic outcomes. Targeting tissue-specific TH actions may result in novel and safe therapeutic options for metabolic dysfunctions.

## Author contributions

AC and DD wrote the manuscript. MD wrote and supervised the manuscript.

### Conflict of interest statement

The authors declare that the research was conducted in the absence of any commercial or financial relationships that could be construed as a potential conflict of interest.
